# Identifying Low Value Care Practices in UK Paediatric Intensive Care Units in 2025: A Delphi Study

**DOI:** 10.1111/nicc.70235

**Published:** 2025-11-09

**Authors:** Lyvonne N. Tume, Lindsay Kenworthy, Emma C. Alexander, Rebecca Mitting, Gerri Sefton, Alison Jones, the Paediatric Critical Care Society De‐Implementation Working group

**Affiliations:** ^1^ Edge Hill University & Alder Hey Children's NHS FT Ormskirk UK; ^2^ Alder Hey Children's NHS Liverpool UK; ^3^ Paediatric Critical Care Research Group Imperial College London London UK; ^4^ Imperial College Healthcare NHS Trust London UK; ^5^ Imperial College London London UK; ^6^ Alder Hey Children's NHS Foundation Trust Liverpool UK; ^7^ Birmingham Children's Hospital Birmingham UK

**Keywords:** child, critical care unit, de‐implementation, intensive care

## Abstract

**Background:**

Low‐value care, is care that is ineffective, inefficiently delivered or unwanted and is common in high income settings. The United Kingdom (UK) Paediatric Critical Care Society (PCCS) established a de‐implementation working group in October 2024.

**Aim:**

The aim of the study was to identify and then prioritise low‐value care practices for de‐implementation across UK Paediatric Intensive Care Units (PICUs) over the next 5 years and to ascertain which practices staff would be prepared to de‐implement based on current evidence, and for which further evidence is required.

**Study Design:**

A modified three round Delphi study was undertaken between December 2024 and May 2025. In Round 1, PICUs submitted their top low‐value care practices. These were entered into an electronic survey (Round 2) for prioritisation. These were analysed according to a predefined consensus definition. The final survey (Round 3), including these items, plus any new items suggested at Round 2, were re‐ranked considering the group mean score.

**Results:**

Thirty‐seven items were submitted from 16 PICUs (these were unit responses) in Round 1. One hundred thirty‐five staff completed Round 2, and 14 of these 37 items met the criteria for inclusion in Round 3. Five new items were added to Round 3. One hundred eighteen staff voted on 19 items in Round 3. The top five practices ranked by mean score were (1) overprescribing of medications and not deprescribing them once not required, (2) fasting children for prolonged periods after extubation, (3) unnecessary use of non‐sterile gloves, (4) continuing hourly observations in ward‐ready patients and (5) taking routine blood tests.

**Conclusions:**

This is the first study to gain consensus on low‐value care practices to be de‐implemented in UK PICUs.

**Relevance to Clinical Practice:**

Low‐value care practices are wasteful, both financially and environmentally, and impact on healthcare professionals' workload. Identification of these practices will enable future work to de‐implement them.

**Impact Statements**

- What is known about this topic
  ○ There is increased awareness and recognition that low‐value care practices are common in intensive care units
  ○ Low‐value care practices are both financially and environmentally wasteful, increase healthcare professionals' workloads (especially nurses) and cause harm to patients.
- What this paper adds
  ○ This is the first study to describe and gain consensus on low‐value care practice in children's intensive care units and the first one from a UK intensive care perspective.

## 1 Introduction and Background

Many practices undertaken in the Paediatric Intensive Care Unit (PICU) lack underpinning evidence [1]. This may lead to a high prevalence of well‐intentioned but ineffective actions that add workload without improving clinical outcomes. Examples of such practices may include repeated blood tests, exposure to unnecessary tests and imaging, and starving children for procedures unnecessarily [2, 3]. These practices can be wasteful in terms of resources and may have a negative environmental impact [4, 5]. These practices are termed low‐value care practices (LVCP) [2, 3]. These have been defined as practices that are ineffective, inefficient and/or for which their benefit does not outweigh their risks [6].

Around 20 000 children a year in the United Kingdom are admitted to PICUs [7] and are thus exposed to these potentially harmful practices without known value. Research increasingly recommends new treatments and practices that should be implemented to improve care, and PICUs therefore attempt to incorporate these into their workflows. However, unless existing practices that are not proven to be effective are de‐implemented alongside the implementation of new practices, the overall workload increases. To improve efficiency, reduce exposure to unnecessary interventions and reduce the negative environmental impact of health care as well as reducing the exposure of patients to potentially harmful or unnecessary care, it is important for the critical care community to identify LVCP and to explore methods for de‐implementation. For some practices, for staff to have confidence that the practice is truly un‐necessary, they will require research evidence; for others, consensus may suffice.

The aim of this study was therefore to identify LVCPs in UK PICUs and prioritise practices for de‐implementation over the next 5 years and to ascertain which practices staff would be prepared to de‐implement based on current evidence, and for which further evidence is required.

## 2 Design and Methods

A modified, three‐round, Delphi study was undertaken between December 2024 and May 2025. This is reported in line with the DELPHISTAR reporting checklist [8].

### 2.1 Setting and Sample

All UK PICU healthcare professionals were invited who were part of the Paediatric Critical Care Society (PCCS) de‐implementation working group were invited to participate. The group had at this time 72 members (nurses, doctors, pharmacists and allied health professionals) from 19/25 UK PICUs. Additionally, for rounds 2 and 3, all PCCS society members (a wider group of professionals representing all UK PICUs, including high dependency units, specialised PIC retrieval services and networks with 1000 members) were invited to participate. In this study, a range of experts working in the field of PIC, was intended with representation from different professional groups was necessary to enable the identification of LVCPs from the perspective of both the professional ordering or prescribing the intervention and those delivering these at the bedside.

### 2.2 Data Collection Tools and Methods

Participants were invited via email and via the PCCS society email. We recruited participants in two ways: via direct email to our working group members and via the PCCS society email list (in an email from the society). For each survey, three email reminders were sent out a week apart to maximise response rates.

Survey rounds: In Round 1 (R1), invited members from each participating PICU identified in in discussion with their PICU, the ‘unit agreed’, top low‐value care practices locally, these were emailed to the study coordinators (LT/LK) or submitted via the new group NHS Futures secure space. These practices were reviewed, duplicates removed, analysed and categorised, before being entered into an electronic survey (Round 2, R2) in Microsoft Forms.

Survey structure: In both Rounds 2 and 3 anonymised surveys generated from the results in Round 1, participants were only also asked three demographic questions: their role, their PICU and years PICU experience. The surveys were structured in a few parts. In the R2 survey, all items were presented under six categories, in separate survey sections, for rating on a 5‐point Likert scale. Below each of these sections, participants were asked whether they perceived that the practice required research evidence to demonstrate the safety of de‐implementation. They were then asked to add any LVCPs that they thought had not been already identified and the three demographic questions. In the R3 survey, the items were listed with the whole group mean score from R2, asking participants to re‐rank items considering the group mean score. New items added at R2 were invited to be ranked, then a separate section asked participants to decide whether they thought an item required research evidence to stop, and ending with basic demographic questions. We did not collect any identifiable member data in the surveys and although we asked participants to complete both rounds of the survey, as we did not collect identifiers, we could not match participants' results from R2 and R3 of the surveys.

Definition of consensus: We used two methods to predefine consensus IN and consensus OUT for each item. Firstly, the mean scores (and standard deviation) of all the items rated in R2 was calculated, and a threshold for items proceeding to R3 was set above this mean (of the mean scores). Next, we calculated the percentage of items scoring 4 and 5 (highest priority) for each item, with a consensus IN definition that 70% responses for an item should be scoring 4 or 5. For consensus OUT, we defined as 70% of the items scoring 1 or 2.

Consensus IN refers an item which met the consensus criteria on being a low‐value care practice, and consensus OUT means that a practice did not meet out predefined criteria for removing completely. For any topics that did not meet the criteria for consensus IN or OUT, the mean score was used.

### 2.3 Ethical and Institutional Approvals

This was a healthcare professional‐only study undertaken through the Professional Society (PCCS); thus, we were only required to gain PCCS Study Group approval for the survey and the study (approval gained in November 2024). Consent for completion of each of the surveys, was gained from members in question one of both R2 and R3 surveys.

## 3 Data Analysis

Round 1 data were analysed qualitatively by two (LT, LK) team members. Practices submitted were reviewed, duplicates removed, then analysed by simple thematic analysis and categorised, before being entered into an electronic survey (R2) in Microsoft Forms. In R2 and R3 surveys, participants rated practices on a 5‐point Likert scale 5 being the highest priority. These data in MS Forms were downloaded into a CSV file. Means and standard deviations (SD) were calculated for each item, as was the percentage of items rated 4 and 5 and the percentage of items rated 1 and 2 (as per our a priori consensus definition). The mean of the mean scores for all items at R2 was calculated and used as the threshold for items going forward into R3. At R3, both mean and SD were calculated as was percentage of items rated 1 and 2 and 4 and 5. This generated a top five LVCPs (by mean score) and identified any items meeting the stricter criteria for consensus IN and consensus OUT.

## 4 Results

**FIGURE 1:**
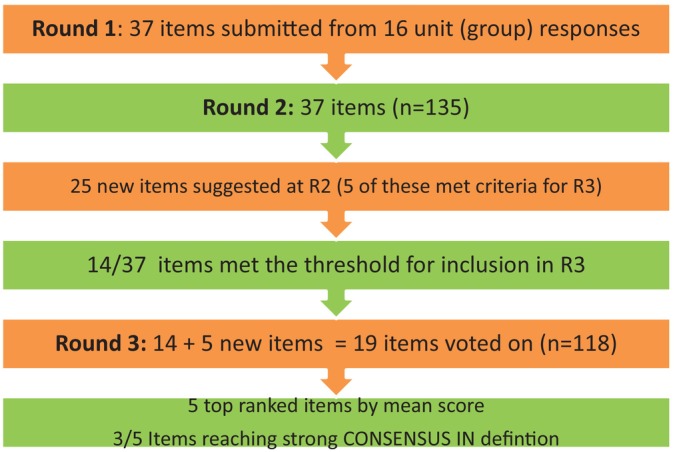
Delphi study flowchart.

Thirty‐seven low‐value care items (categorised under six themes) were submitted from 16/25 PICUs (unit‐agreed responses) in R1. One hundred thirty‐five staff completed R2 (from 23/25 PICUs), and the mean score of all the items was 3.20, with 14 items meeting the criteria for inclusion in R3 (> 3.20). Twenty‐five new items were suggested at R2, of which, after a group meeting and discussion, only five were progressed into R3. The reasons for exclusion were similar and duplicate items, or the new item being a low‐value care practice. Thus, 19 items went into the R3 survey. One hundred eighteen staff completed R3 (from 20/25 PICUs), and consensus (by mean score) was achieved on 14/19 items. Using the stricter consensus IN definition, however, only three of these 14 items met the criteria for consensus IN. No items achieved consensus OUT. Nevertheless, we present the top five ranked items by mean score, highlighting the three of these with the highest degree of consensus (Figure 1, Table 1).

**TABLE 1:** Delphi respondents.

Round 1 Dec 2024–Jan 2025	Responses
Participating PICUs were asked to submit a group response for top low value care practices in their unit	16 PICUs submitted topics 137 topics suggested
Round 2 March–April 2025	*N* = 135
Survey sent out via PCCS membership and via working group	*N* (%)
Role	
Staff nurse (Band 5)	25 (19%)
Senior Staff nurse (Band 6)	13 (10%)
Charge nurse (Band 7)	16 (12%)
Grid trainee/registrar/fellow	10 (7%)
Consultant	30 (22%)
Dietitian	4 (3%)
Physiotherapist	6 (4%)
Pharmacist	7 (5%)
Occupational therapist	0
Other (ACP or research nurses)	24 (18%)
Years PICU experience	8% < 12 months; 11% 1–5 years; 19% 5–10 years; 61% > 10 years
Round 3 May–June 2025	*N* = 118
Survey sent out via PCCS membership and via working group	
Role	
Staff nurse (Band 5)	6 (5%)
Senior Staff nurse (Band 6)	16 (14%)
Charge nurse (Band 7)	17 (14%)
Grid trainee/registrar/fellow	10 (8%)
Consultant	36 (31%)
Dietitian	3 (3%)
Physiotherapist	3 (3%)
Pharmacist	8 (7%)
Occupational therapist	0
Advanced Clinical Practitioner	10 (8%)
Other (Research nurses/ECMO specialist)	9 (8%)
**Years PICU experience**	2% < 12 months; 15% 1–5 years; 26% 5–10 years; 57% > 10 years

The top five practices (by mean score) with a priority to de‐implement over the next 5 years were (1) the overprescribing of medications and not de‐prescribing them once no longer required, (2) fasting children for prolonged (routine time‐designed) periods after extubation, (3) the unnecessary use of non‐sterile gloves, (4) continuing hourly observations in patients waiting discharge to a general ward and (5) taking routine (daily) blood tests. The items in bold met the stricter criteria for consensus IN (Table 2).

**TABLE 2:** Delphi results.

Low‐value care practice	Mean score at R2 (*n* = 135)	Ranked by mean (SD) score at R3 (*n* = 118)	By consensus IN (70% of scores 4 and 5)	Perceived to need research evidence to stop
***Overuse of medications and not stopping them in a timely manner***	3.75 (1.37)	4.13 (1.02)	**Consensus IN**	59% No research required
***Prolonged fasting after extubation***	3.67 (1.40)	4.0 (0.99)	**Consensus IN**	36% YES 34% NO 30% unsure and
***Unnecessary use of non‐sterile gloves***	3.67 (1.66)	3.94 (1.15)	Not consensus IN or OUT	44% No research required 34.7% YES 22% unsure
***Continuing hourly observations on patients identified as ‘ward‐ready’***	NA added in R2	3.90 (1.20)	Not consensus IN or OUT	87% No research required
***Taking routine (daily) blood tests***	3.68 (1.51)	3.87 (1.16)	**Consensus IN**	67% No research required
Taking blood gases without a clear clinical indication	3.58 (1.46)	3.86 (1.14)	Not consensus IN or OUT	73% NO research required
Routine central line changes prophylactically if no signs of infection	NA added at R2	3.70 (1.20)	Not consensus IN or OUT	57% YES research required
Fasting for most PICU procedures	3.42 (1.53)	3.65 (1.16)	Not consensus IN or OUT	45% No; 32% YES 23% unsure
Wearing a plastic apron to examine a child	3.51 (1.57)	3.57 (1.40)	Not consensus IN or OUT	78.8% NO research required
Fasting for 4–6 h before extubation	3.34 (1.34)	3.30 (1.19)	Not consensus IN or OUT	65.3% YES
Changing dressings if they are intact with no signs of infection	NA added at R2	3.29 (1.23)	Not consensus IN or OUT	37.1% YES (49% No just stop)
Other high‐ranking items at R3 removed with rationale				
Duplication of documentation across IT systems		3.96	Discussed with group, not viable as not within control of the PICU. Needs addressing at organisational level.	
Opening new vials of drugs for each child when they could be shared		3.92	Consensus by UK PICU pharmacists' group this practice not viable due to risk of contamination	
Changing intravenous drug infusions every 24 h if the drug is stable		3.80	Consensus by UK PICU pharmacists' group this practice not viable due to risk of contamination	

*Note:* No items achieved consensus OUT in R3. Green coloured are the top 5 priority areas, white the next 5 and the zone in purple is items that ranked highly but had to be removed with the rationale.

Abbreviations: IT, Information Technology; R2, Round 2; R3, Round 3; RCT, randomised controlled trial; SD, Standard deviation.

## 5 Discussion

This is the first UK and paediatric intensive care study to identify and gain consensus on low‐value care practices. A similar international campaign was launched in North America in 2012, the ‘Choosing Wisely’ campaign, in response to the soaring healthcare costs [9], the need to improve the quality of clinical care delivered and avoid waste. These recommendations were enthusiastically adopted by the North American adult critical care community, endorsed by the Society of Critical Care Medicine [9]. These campaigns, updated at key intervals, identify the top five recommendations of low‐value care practices and direct clinicians to make better decisions. The latest (2021) recommendations prioritise five areas about which to ‘choose wisely’ including unnecessary catheter usage, delaying liberation from mechanical ventilation, continuing antibiotic therapy without need, delaying mobilisation and providing care discordant to the patient's goals and values [10]. These broadly differ from the top LVC practices in UK PICUs, with only one ‘not continuing antibiotic therapy without evidence of need’, inclusive of our broader ‘overuse of medications and not stopping them’ item.

Similarly, the EVOLVE programme led by the Royal Australasian College of Physicians supports staff to safely reduce low‐value tests, treatments and procedures where appropriate, to provide high‐value care to patients based on evidence and expertise and influence the best use of health resources, reducing wasted expenditure [11]. In 2016, they identified low‐value care practices in paediatrics to be de‐implemented [12] relating to unnecessary imaging, unnecessary prescribing amongst others, but none of these relate to PICU practice. The European Academy of Paediatrics recommended reducing overtreatment and low‐value care practices in a 2019 statement [13].

In the United Kingdom, however, no intensive care society has, until now, undertaken a study specifically to identify and gain consensus on low‐value care practices across UK ICUs. Although the UK (adult) Intensive Care Society has a sustainability agenda and has endorsed the ‘Gloves off’ campaign to reduce the unnecessary use of unsterile gloves, and although ICS supports the de‐implementation of low‐value care practices, they have not specifically identified or recommended any beyond reducing unsterile glove usage [14]. This is important because intensive care units (of all types) are high resource, risk‐averse environments where clinicians are a self‐selected population with a preference for action over inaction [15].

Our study is novel because, firstly, it is the only UK‐based ICU study, it is also the only study specific to paediatric ICUs and it is the only study to include wider healthcare professionals (beyond physicians) in identifying low‐value cares, thus our findings have broader scope compared to others. Two of our top five items have not been previously identified—that of prolonged fasting after extubation and continuing hourly observations (impacting on nursing time) on children ready for ward discharge, but in whom discharge is delayed (a frequent occurrence in PICUs).

## 6 Limitations

There are several limitations of this study that warrant acknowledging. Firstly, items in the first round of the study were generated by only 16 PICUs (out of 25 level 3 PICUs in the United Kingdom). However, in subsequent rounds we had respondents from most UK PICUs, and any items felt to be missing could be added. Secondly, aside from the working group members, only members of PCCS were invited to participate, and this may not represent the wider healthcare professionals working in UK PICUs. Although we sought to capture respondents across multiple roles and seniorities, respondents may reflect those who already have views about de‐implementation by nature of the optional, survey‐led work. Furthermore, although we intended to only seek the views of PICU staff, broader more objective views from those involved in but external to PICU (e.g., microbiologists) may have provided different results.

Despite these limitations, however, we had a reasonable response rate, low attrition between rounds and good representation from different disciplines and grades of staff from across 24/25 UK PICUs.

## 7 Conclusions

UK PICU staff recognise that some current care practices are low in value. Our study is the first to identify and prioritise these and the first UK‐based ICU study in this field involving all PICU healthcare professionals. Identifying and gaining consensus on low value care in UK PICUs is the first step in the PCCS's efforts to reduce these, with a focus on providing quality care, reducing costs, waste and environmental impact and to improve safety and quality of life in children and their families.

## Author Contributions

L.T. conceived the study, but all authors listed were actively involved in the study (L.T. and L.K. analysed the data) and have contributed to and reviewed the manuscript.

## Ethics Statement

This study was approved by the UK PCCS Society and involved only staff.

## Conflicts of Interest

The authors declare no conflicts of interest.

## Supporting information


**Data S1:** Round 2 Delphi survey.

**Data S2:** Round 3 Delphi survey.

## Data Availability

The data that support the findings of this study are available from the corresponding author upon reasonable request.
