# Supplementary material for: Identifying Low Value Care Practices in UK Paediatric Intensive Care Units in 2025: A Delphi Study
Source: Nurs Crit Care. 2025 Nov 9;30(6):e70235. doi: 10.1111/nicc.70235 (PMC12598117; doi:10.1111/nicc.70235)
Supplement: Supplementary file 1 — Data S1: Round 2 Delphi survey. [file NICC-30-0-s001.docx]

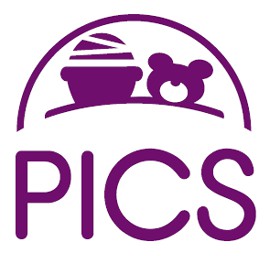


PCCS Prioritizing low value care practices in UK Paediatric Critical Care Units Round 3

This is the 2nd survey in which you will be asked to re-rate the importance of the top scoring low value care practices from Round 2 in light of the groups mean score. We ask you to think on national level now (not just for your unit). We

want to get consensus on the top 5 practices that need to be de-implemented in UK PCCUs over the next 2-5 years, and the top 5 areas requiring research so please don't rate them all high, think about the ones that are the TOP priority at a national level. **Please rate each practice on the 1-5 scale where 5 is the HIGHEST priority (most important to stop nationally).**

The survey will take around 10 minutes to complete.

Because there are people with different roles (physios, nurses, doctors, pharmacists, dieticians) completing this survey

and some may not be able to rate some of the practices, we have not made the rating of each question mandatory, ***so if you do not know about a practice, simply don't answer that question***.

Some of these practices can just be stopped (don't require research) and others will need research to show it is safe to

stop them. After rating these, you will be asked which of these (if any) you believe requires evidence to show it is safe to stop doing it. So what practice/s would you be happy to stop now? If you don't know simply write NA or DK (Don't

know).

Finally, any non-duplicate low value care practices that you suggested in the Round 2 survey have been added to the list to rate (they wont have a group mean score). Finally some basic (non-identifiable) demographics (unit, main role and

grade and years PICU experience) will be collected.

The results of this study will be presented at PCCS conference and feed into a PCCS Position Statement.

This study is led by Professor Lyvonne Tume on behalf of the PCCS De-implementation working group, if you have any questions please email [lyvonne.tume@edgehill.ac.uk](mailto:lyvonne.tume@edgehill.ac.uk) and the study has been approved by the PCCS Study group.

* Required

# Do you consent to complete this survey? *


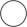

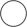
 Yes No

# Please re-rate (considering the group mean score) how important it is to stop these practices (on a national level). A rating of 5 is the highest priority to stop, scoring 1 means it is not

important to stop this. Below are the 14 top rated low value care practices in descending order from from the first survey.

| 1 | 2 | 3 | 4 | 5 |
| --- | --- | --- | --- | --- |


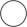

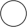

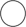

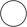

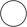
Opening new vials of drugs for each child (when they

could be shared) **mean score 3.84**

Overuse of medications and not

**
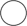

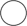

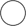

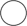

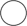
**stopping them in a timely manner **mean score 3.75**


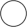

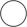

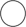

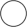

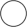
Routine (daily) blood

tests **mean score 3.68**


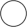

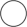

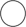

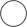

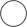
Use of non- sterile gloves

unnecessarily **m ean score 3.67**

Prolonged fasting

**
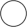

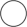

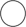

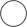

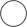
**after extubation **mean score 3.67**


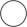

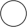

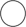

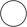

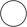
Duplication of documentation across IT

systems **mean score 3.63**


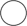

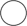

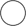

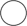

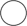
Taking blood gases without clear clinical

indication **mea n score 3.58**

**
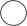

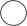

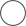

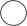

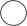
**Changing drug infusions 24h if stable **mean**

**score 3.57**

**
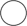

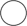

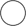

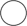

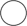
**Wearing a plastic apron to examine a child **mean score 3.51**

Fasting for most PICU

**
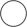

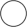

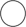

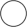

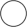
**procedures (in intubated children) **mean score 3.42**

Fasting 4-6hrs before

**
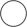

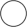

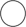

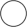

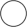
**extubation **mean score 3.34**

**
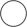

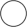

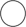

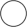

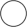
**Omeprazole for patients NBM **mean score 3.24**

1 2 3 4 5

Minimal/low


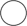

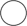

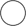

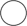

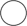
thresholds for taking

cultures **mean score 3.23**

# Please rate these 'new' low value practices suggested from the first survey. Your rating

means how important it is to stop these practices (on a national level now), with a score of 5 is the most important to stop, scoring 1 means it is not important to stop this.

| 1 | 2 | 3 | 4 | 5 |
| --- | --- | --- | --- | --- |

Hourly


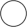

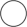

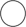

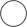

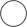
observations on dischargeable

patients once identified at

‘wardable’

Changing dressings if


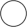

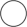

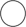

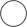

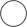
they’re intact

without signs of infection

Routinely


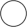

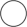

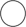

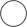

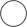
muscle relaxing children for

transport

Routine CVL


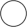

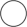

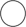

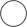

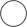
changes every 'x' days if no

signs of sepsis (& access difficult)

Daily sheet


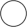

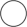

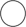

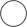

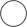
change when they are clean

# For the 14 top rating low value care practices in question 2, please indicate those that do and those that do not require research to show they are safe to stop

| Yes needs research to show it is safe to stop | No we should just stop this | I'm not sure |
| --- | --- | --- |


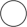

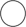

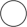
Fasting (4-6 hours) before extubation


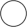

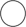

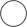
Prolonged (4 hrs) fasting after extubation

Fasting for most PICU


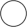
procedures (in already

intubated patients)

Changing drug infusions 24

hourly if stable

Omeprazole for patients NBM

Overuse of medications and not

stopping them in a timely manner

Opening new vials of drugs for each child (when they

could be shared)

Taking blood

gases without a clear clinical

indication

Routine (daily) blood tests

Minimal/low

thresholds for taking cultures

Wearing a plastic apron to examine a child

Use of unsterile gloves

uneccesarily

Duplication of documentation across IT

systems

# For the 5 new low value care practices please indicate those that do and those that do not require research to show they are safe to stop

| Yes needs research to show it is safe to stop | No we should just stop this | I'm not sure |
| --- | --- | --- |

Hourly

observations on dischargeable

patients once identified at

‘wardable’

Changing dressings if

they’re intact

without signs of infection

Routinely

muscle relaxing children for

transport

Routine CVL

changes every 'x' days if no

signs of sepsis (& access difficult)

Daily sheet

change when they are clean

About you, we need to collect some non-identifiable data about you

# Which Paediatric Critical Care Unit or transport service are you from? *

1. What is your main role *

Staff nurse (band 5)

Junior charge nurse/ Band 6

Senior charge nurse/clinical manager (band 7) PICU trainee/grid/registrar/fellow

Consultant Dietician

Physiotherapist Pharmacist

Occupational Therapist

Advanced Clinical Practitioner Other

# How many years Paediatric Critical Care Experience do you have? *

<12 months

1-4.9 years

5-10 years

>10 years

This content is neither created nor endorsed by Microsoft. The data you submit will be sent to the form owner.

Microsoft Forms
