# Supplementary material for: Identifying Low Value Care Practices in UK Paediatric Intensive Care Units in 2025: A Delphi Study
Source: Nurs Crit Care. 2025 Nov 9;30(6):e70235. doi: 10.1111/nicc.70235 (PMC12598117; doi:10.1111/nicc.70235)
Supplement: Supplementary file 2 — Data S2: Round 3 Delphi survey. [file NICC-30-0-s002.docx]

PCCS Prioritising low value care practices in UK Paediatric Critical Care Units Round 2


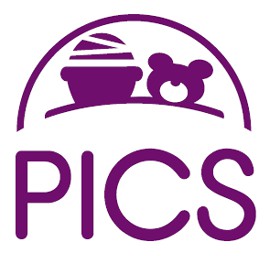


This is 1 of 2 surveys for you to rate the importance of previously identified (by 16 units) low value care practices in UK PCCUs. We want to get consensus on the top 5 practices that need to be de-implemented over the next 2-5 years, and the top 5 areas requiring research so please don't rate them all high, think about the ones that are the TOP priority at a national level. **Please rate each practice on the 1-5 scale where 5 is the HIGHEST priority (most important to stop nationally).**

The survey will take around 10 minutes to complete.

Because there are people with different roles (physios, nurses, doctors, pharmacists, dieticians) completing this survey

and some may not be able to rate some of the practices, we have not made the rating of each question mandatory, ***so if you do not know about a practice, simply don't answer that question***.

Some of these practices can just be stopped (don't require research) and others will need research to show it is safe to

stop them. After rating these, you will be asked which of these (if any) you believe requires evidence to show it is safe to stop doing it. So what practice/s would you be happy to stop now? If you don't know simply write NA or DK (Don't

know).

You will also be asked (at the end) if there are any things you think are missing from this list. These practices are

grouped within 6 main themes. Finally some basic (non-identifiable) demographics (unit, main role and grade and years PICU experience) will be collected.

In the 2nd survey to follow in around 6 weeks time, you will see how the group rated the practices and be asked to re- rate the topics considering the group scores.The results of this study will be presented at PCCS conference and feed into a PCCS Position Statement.

This study is led by Professor Lyvonne Tume on behalf of the PCCS De-implementation working group, if you have any questions please email [lyvonne.tume@edgehill.ac.uk](mailto:lyvonne.tume@edgehill.ac.uk) and the study has been approved by the PCCS Study group.

* Required

1. Do you consent to complete this survey? *


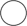

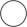
 Yes No

1. Please rate these low value care practices around routine changing of equipment (rating 5 is highest priority)

| 1 | 2 | 3 | 4 | 5 |
| --- | --- | --- | --- | --- |


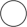

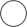

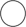

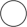

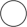
Changing ventilator

circuits weekly


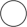

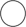

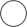

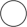

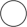
Changing in- line suction

catheters daily


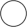

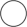

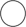

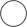

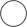
Suction tubing changed daily


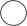

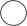

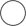

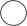

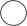
Weekly change of urinary catheter bags

Changing arterial flush


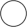

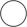

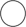

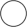

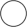
solutions (bag & set) every 48- 72hours


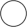

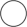

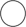

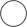

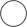
Changing the ventilator filter daily

1. Of these practices listed above which (if any) do you believe require research evidence of safety to stop them? *
2. Please rate these low value care practices related to nutrition (rating 5 is the highest priority)

| 1 | 2 | 3 | 4 | 5 |
| --- | --- | --- | --- | --- |


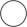

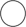

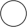

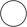

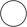
Fasting (4-6 hours) before extubation


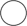

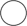

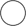

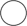

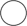
Prolonged (4 hrs) fasting after

extubation

Fasting for most PICU


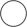

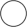

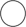

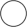

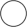
procedures (in already

intubated patients)

Gastric


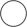

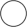

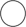

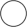

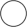
aspiration pre- feeds


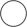

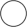

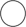

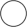

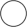
Stopping the lipids (in PN) for 4 hours

daily pre bloods

1. Of these practices listed above which (if any) do you believe require research evidence of safety to stop them? *
2. Please rate these low value care practices related to medications (priority of those you think require research to stop or should just be stopped)

| 1 | 2 | 3 | 4 | 5 |
| --- | --- | --- | --- | --- |

3 months


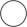

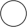

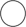

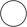

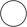
heparin post line DVT


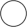

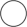

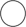

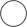

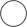
Giving a test dose of

clonidine


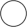

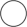

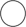

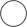

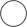
Double RN checking of drugs


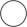

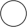

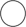

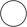

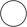
Changing drug infusions every 24 h if drug

stable


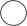

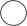

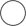

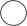

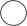
Dexamethasone prior to

extubation


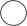

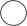

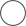

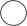

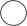
Sedation prior to ETT tape

changes


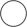

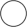

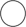

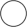

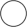
Omeprazole for NBM patients

Pharmacologica l DVT


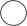

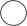

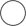

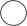

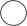
prophylaxis in immobilised children

Overuse of certain


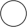

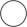
medications and not

stopping them

Opening new vials of drugs for each

patient, when could be

shared

1. Of these practices listed above which (if any) do you believe require research evidence of

safety to stop them? or are there are technical and regulatory issues that need considering (please detail these) *

1. Please rate these low value care practices related to bloods and laboratory tests

| 1 | 2 | 3 | 4 | 5 |
| --- | --- | --- | --- | --- |

Unnecessary

blood gases to check or post vent change etc

Unnecessary blood tests

(routine daily bloods)

Minimal/low

thresholds for 'cultures'

1. Of these practices listed above which (if any) do you believe require research evidence of safety to stop them? *
2. Please rate these low value care practices relating to infection control (5 is highest)

| 1 | 2 | 3 | 4 | 5 |
| --- | --- | --- | --- | --- |

Wearing a plastic apron to examine a child

Use of non- sterile gloves unnecessarily

Single use of breast feeding

packs for same mother

Swabbing of IV sites weekly

Routine Resp

secretions sent 3 x week and at admission

/weekly

1. Of these practices listed above which (if any) do you believe require research evidence of safety to stop them? *
2. Please rate these low value care practices relating to imaging (5 is highest)

| 1 | 2 | 3 | 4 | 5 |
| --- | --- | --- | --- | --- |

Routine CXRs on admission

Routine CXRs after chest

drain removed in cardiac surgical kids

routine CXR after ETT re- positioning

Daily CXR for children on HFOV

1. Of these practices listed above which (if any) do you believe require research evidence of safety to stop them? *
2. Please rate these low value care practices relating to endotracheal suction and ETTs

| 1 | 2 | 3 | 4 | 5 |
| --- | --- | --- | --- | --- |

Instillation of

saline routinely for suction

Use of sterile gloves for endotracheal suction

Routine 4 hourly cuff

pressure check and deflation

Suctioning

routinely every 4 hours

1. Of these practices listed above which (if any) do you believe require research evidence of safety to stop them? PS Three of these already have robust evidence *
2. Please rate these miscellaneous low value care practices

| 1 | 2 | 3 | 4 | 5 |
| --- | --- | --- | --- | --- |

Intentional rounding

Daily urinalysis dipstick

Duplication of documentation across different IT platforms

1. Of these practices listed above which (if any) do you believe require research evidence of safety to stop them? *
2. **Please describe any any practice/s you believe are low value** (ie add nothing to the care we deliver and are potentially unnecessary) **that have not already been identified.** Please don't add things we SHOULD be doing, this survey is about things we are doing that we

SHOULD NOT be doing, that are potentially wasting time, resources and increasing staff workload *

About you, we need to collect some non-identifiable data about you

1. Which Paediatric Critical Care Unit or transport service are you from? *
2. What is your main role *

Staff nurse (band 5)

Junior charge nurse/ Band 6

Senior charge nurse/clinical manager (band 7) PICU trainee/grid/registrar/fellow

Consultant Dietician

Physiotherapist Pharmacist

Occupational Therapist Other

1. How many years Paediatric Critical Care Experience do you have? *

<12 months

1-4.9 years

5-10 years

>10 years

1. We thank you for taking the time to complete this first survey. Is there anything else you want to tell us about low value care practices in paediatric critical care units in the UK?

This content is neither created nor endorsed by Microsoft. The data you submit will be sent to the form owner.

Microsoft Forms
